# PEEK versus Metallic Attachment-Retained Obturators for Patient Satisfaction: A Randomized Controlled Trial

**DOI:** 10.1055/s-0041-1731839

**Published:** 2021-11-23

**Authors:** Mohamed Yahia Sharaf, Asharaf Email Eskander

**Affiliations:** 1 Department of Prosthodontics, Faculty of Dentistry, University of Menoufia, Menoufia, Egypt; 2 Department of Prosthodontics, Faculty of Oral and Dental Medicine, University of Cairo, Cairo, Egypt

**Keywords:** attachment, maxillectomy, obturator, patient satisfaction, PEEK

## Abstract

**Objective** The aim of the study was patients’ satisfaction evaluation and radiographic evaluation of the terminal abutments of attachment-retained maxillary obturators with metal framework versus milled polyetheretherketone (PEEK) framework in the management of maxillectomy cases.

**Materials and Methods** Eighteen participants were randomly divided into three parallel groups ( *n* = 6). Participants of the PEEK group received attachment-retained obturators with milled PEEK framework, the metal group received an attachment-retained obturator with a metallic framework, and the conventional group received conventional clasp-retained obturators with a metallic framework (Control group). The evaluation included was radiographic evaluation and patients’ satisfaction in this study included two scales—”The Obturator Functioning Scale” and “The European Organization for Research and Treatment of Cancer Head and Neck 35” using one-way ANOVA test.

**Results** Both PEEK and metal groups showed a statistically significant lower mean bone loss ( *p* <0.050) compared with the conventional group during all follow-up periods. There is no statistically significant difference between the PEEK and metal groups during all follow-up periods. Regarding patient satisfaction, both the PEEK and metal groups showed a statistically significant decrease score ( *p* <0.050) compared with the conventional group in various aspects of patients’ satisfaction scales as satisfaction with the look and difficulty of talking to the public, and noticeable clasps. In comparison, the PEEK group showed a statistically significant decrease score ( *p* <0.050) than the metal group with respect to satisfaction with the look along all follow-up periods.

**Conclusions** PEEK attachment-retained maxillary definitive obturators could be considered a promising treatment modality for patients with acquired maxillary defects with regard to esthetics and satisfaction.

## Introduction

Prosthetic rehabilitation of acquired maxillary defects represents a challenging mission for both the prosthodontist and psychologically traumatized patients. These defects may be due to trauma, pathological conditions, or surgical resection of oral tumors. The resulting main problem is oronasal communication leading to impairment in mastication, swallowing, speech, and facial esthetics. 1 2 3 Where an ideal treatment planning and design leading to satisfactory prosthetic rehabilitation of these maxillary defects. 4 The primary aim of prosthetic rehabilitation is the closure of oronasal communication to prevent hypernasal speech, fluid leakage into the nasal cavity, improve masticatory function, swallowing, speech intelligibility, articulation, restore facial contours, and improve both quality of life and patient satisfaction. 5 6 7 8 9 10 However, rehabilitation of unilateral maxillary resection has intrinsic leverages that act as dislodging factors. 1 2 3 Therefore, the rehabilitation of maxillary defects represents a challenging task with regard to preserving of precious remaining structures, creating retention, stability, and overcoming the functional stresses. 11 Besides, the weight of the obturator is a critical point and should be kept as minimum as possible to counteract the effect of gravity. 12 This could be achieved by constructing a hollow bulb obturator with or without a top, using a sectional obturator, or utilizing lightweight material. 13 14 15 16 Rests, and vertical guiding planes may provide support and stability for the obturator. 12 Furthermore, the use of soft lining material for the defect may enhance the patient’s quality of life and comfort as it is flexible and protects the integrity of the adjacent moving tissues. 17 Surgical reconstruction of maxillectomy defects is not always possible despite the recent advances in the maxillofacial surgical field, which may be due to poor patients’ general health, advanced age, and large defects. 18 19 Also, bone grafting is not recommended because the blood supply to the graft area is compromised, especially after radiotherapy. Also, the ability to follow-up the recurrence of the tumor is not applicable, as well as accumulation of mucous and nasal discharge on the nasal side of the flap cause unpleasant odors and local infections. 20 The use of dental implants and zygomatic implants through the maxillary sinus in the intact side may provide an adequate bone quantity and quality for implant placement. Thus, a high level of functional rehabilitation can be achieved by using dental implants. Unfortunately, these anchorage sites are often limited because of aggressive tumor resection area, excessive tissue loss, or compromised tissue beds due to radiation. 11 21 22 23 24 25 Mainly, most maxillary defects are rehabilitated with a conventional obturator that uses various clasps as retentive means. 26 27 However, the use of various types of attachments may be of value in improving retention and stability of the prosthesis, as well as improving the seal leading to water and airtightness. Attachments are superior to conventional clasping for improving esthetics, retention, as well as quality of life, especially if incisors are terminal abutments and adjacent to a large defect. 7 28 29 30 31 It was reported that the prosthetic rehabilitation of a maxillary defect with an obturator retained by extracoronal resilient attachments could be of value in conserving tooth structure and satisfying both esthetic and functional aspects. 32 Besides, it helped in achieving stability. It reduced the leverage for the remaining teeth adjacent to the defect. 1 Recently, the need for knowledge of the multidimensional impact of maxillofacial tumors on a patient’s life has led to an increased need to evaluate the quality of life and patient satisfaction. A satisfactory obturator significantly contributes to improving psychological well-being and the quality of life for maxillectomy patients. 33 Thus, with the introduction of new materials that could improve the prosthetic appliances as PEEK (polyetheretherketone), which represents a high-performance thermoplastic polymer of high hardness and elastic modulus ranges between 3 and 4 GPa. PEEK is characterized by lower water absorption, solubility, and nearly no biofilm formation, which is a critical factor for the hygienic nature of prostheses, especially in the maxillofacial prosthesis. PEEK is equal to or better than titanium in biofilm formation. Therefore, PEEK is an exciting alternative to traditional alloys. 34 35 36 PEEK is a biocompatible material that has been used widely in the prosthetic field as implants, provisional abutments, implant-supported bar, clamp material, bridges, or crowns. 37 38 39 40 There are two methods for PEEK processing either milling out of blanks using computer-aided design/computer-aided manufacturing (CAD/CAM) or vacuum pressing. 41

The purpose of this clinical trial was to compare patient satisfaction of the attachment-retained CO-CR versus PEEK-retained maxillary obturators. The research hypothesis was that the PEEK-retained maxillary obturator will provide better patient satisfaction.

## Materials and Methods

Eighteen participants (six females and 12 males) wearing interim or conventional obturators were selected according to the following criteria: participants with a sufficient number of natural teeth (class I and/or class IV Aramany classification) not less than five teeth, mouth opening is not less than 25 mm, intact soft palate, and participants were not exposed to radiotherapy or chemotherapy in the previous year. Participants were randomly divided (sealed envelopes technique) into three parallel groups ( *n* = 6), and the allocation concealment at the department’s chairman. All included participants agreed to have the treatment and signed the informed consent. The study was approved by the Ethical Committee and adhered to the principles of the Declaration of Helsinki. The study was registered at Clinical trials.gov (NCT04778254).

Sample size calculation: Based upon the results of Chen et al, 42 using alpha (α) level of 5% and beta (β) level of 10%, i.e., power = 90%, the study will include a minimum of six subjects per group for a total of 18 subjects. Sample size calculation was performed using IBM SPSS Sample Power Release 3.0.1.35.

PEEK group: Participants of this group received attachment-retained obturators of CAD-CAM-milled PEEK framework.

Metal group: Participants of this group received an attachment-retained obturators of (cobalt chromium) CO/CR framework.

Conventional group: Participants of this group received a clasp-retained obturators.

Maxillary and mandibular alginate impressions (Hydrogum 5, Zhermack S.p.a., Badia Polesine, Rovigo, Italy) were made (after modification of the upper stock tray) and poured into dental stone to obtain the study. Teeth preparation and temporary crown construction were performed. In a sectional stock tray, an impression of the remaining teeth before preparation was made using rubber base impression materials (ZetaPlus, Zhermack S.p.a., Badia Polesine, Rovigo, Italy) to construct temporary crowns. The natural teeth on the intact side were reduced and prepared with subgingival finishing lines to be ready for crowning except for the wisdom tooth if it is present. The final impression was made using a rubber base; temporary composite crowns were made, finished, polished, and cemented temporarily.

**Fig. 1:**
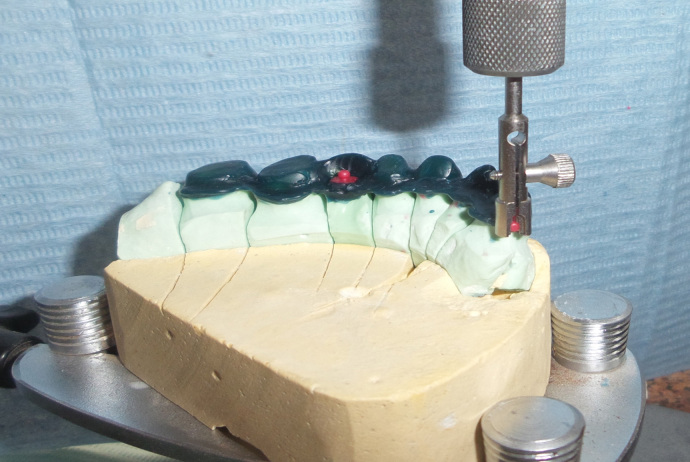
Wax pattern at the mesiopalatal surface of the most anterior abutment

After obtaining the master cast, sawing of the cast and dowel pin placement were performed. A face bow record was made for mounting the maxillary cast on a semiadjustable articulator. An interocclusal wax record was performed for mounting the mandibular cast. A wax pattern for the splinted crowns was performed. A ledge was made on the palatal surface of the wax pattern using a milling machine (Milling machine, Bredent Company). The OT strategy attachments (Rhein 83) were used in these cases where the first one was attached to the wax pattern at the junction of the distopalatal area of the second premolar and mesiopalatal of the first molar (in some cases with missed first molar, the attachment placed at the site of the missed molar). The second one was attached to the wax pattern at the mesiopalatal surface of the most anterior abutment (► Fig. 1 ). Investing and casting the wax pattern were done. Then, the metal try-in of the splinted crowns and construction of the splinted crowns were completed. Then the splinted crowns with attachment were checked inside the patient’s mouth for proper occlusion. For definitive obturator construction, an overall impression was made while the splinted crowns inside the patient mouth and poured in to extra hard dental stone. Arbitrary block out was done to block any undesirable undercut on the stone cast with clay. The clips were also placed over the attachment.

**Fig. 2:**
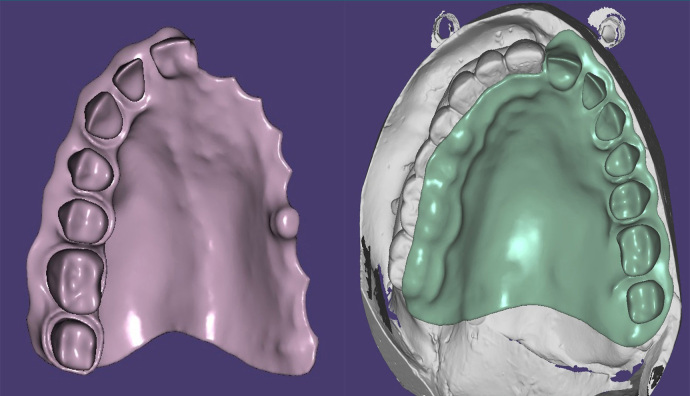
Design performed on dental software (Exocad GmbH, Darmstadt, Germany) to produce an STL file.

**Fig. 3:**
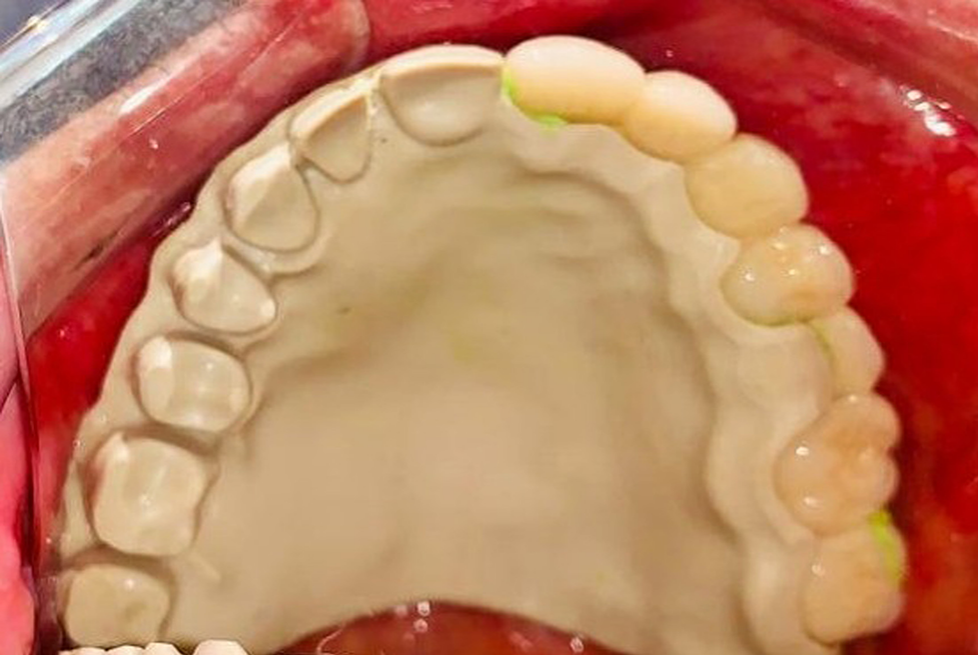
Try in of the PEEK framework. PEEK, polyetheretherketone.

**Fig. 4:**
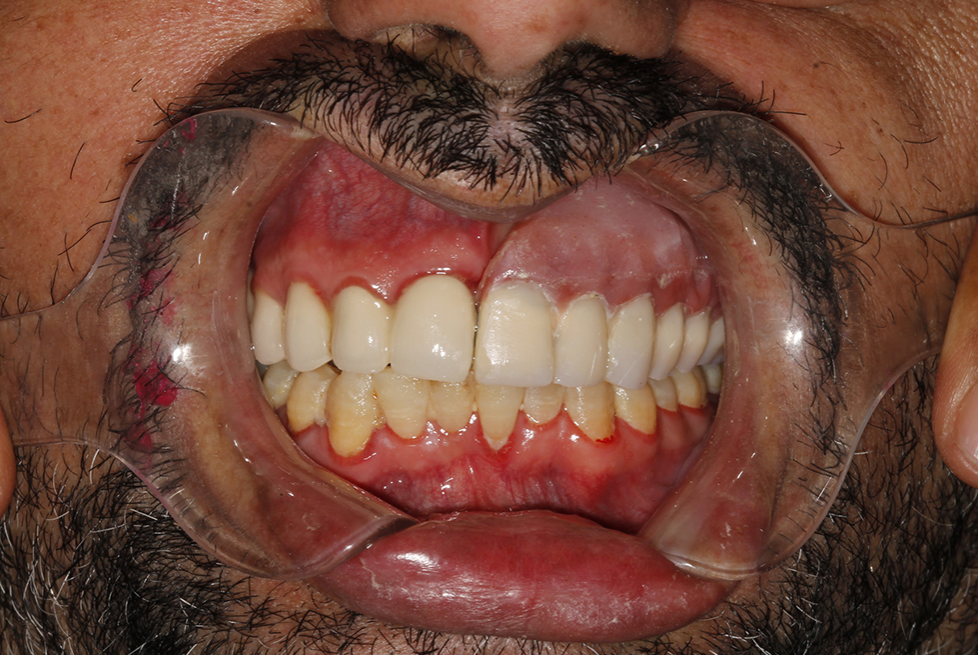
Final PEEK obturator. PEEK, polyetheretherketone.

PEEK group: PEEK blanks with a dimension of 98.5 × 23 mm (Dental Direkt GmbH, Spenge, Germany) were used to produce the obturator frameworks. A laboratory scanner scanned the blocked-out cast to obtain an (standard tessellation language) STL file. The design was performed on dental software (Exocad GmbH, Darmstadt, Germany) to produce an STL file transferred to the CAM Software to be milled on the five-axis milling machine (► Fig. 2 ). After finishing the prosthesis, the retentive clips were placed using a clip inserting tool inside the peek housing. However, two clips were cemented (Calibra Universal Dual Cure AutoMix, Dentsply) for fixation inside the housing as they were loose. The framework was tried in the patient’s mouth and checked for adaptation and extension ( Fig. 3 ). Building up of teeth and gingival form using Viso.lign and Crea.lign (Bredent GmbH & Co. KG, Senden, Germany) was performed and tried in the patient’s mouth ( Fig. 4 ). Occlusal adjustments were performed intraorally, then the obturator finished and polished.

**Fig. 5:**
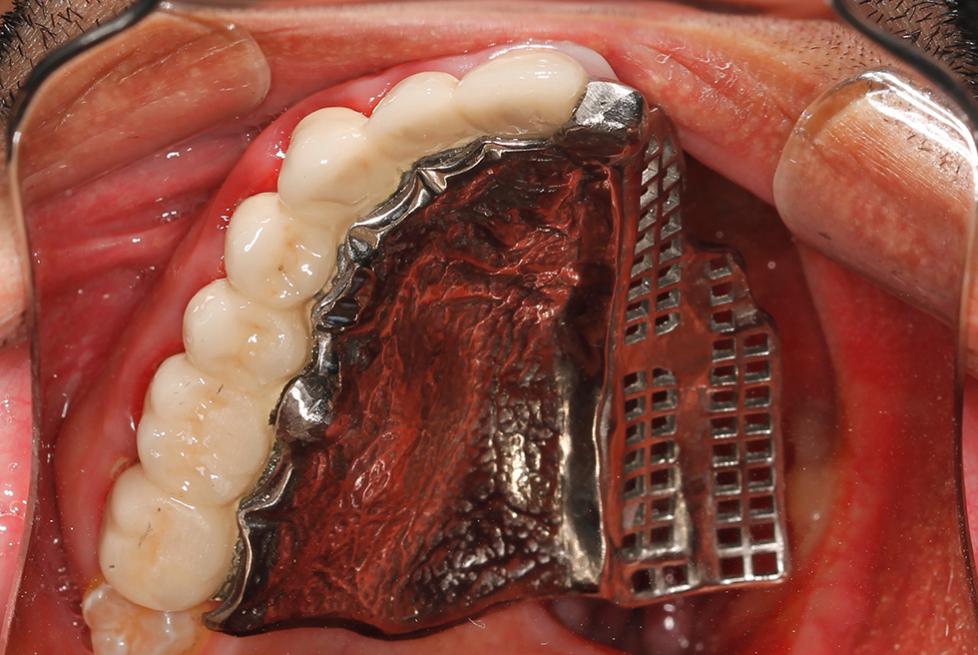
Try in of metal framework.

**Fig. 6:**
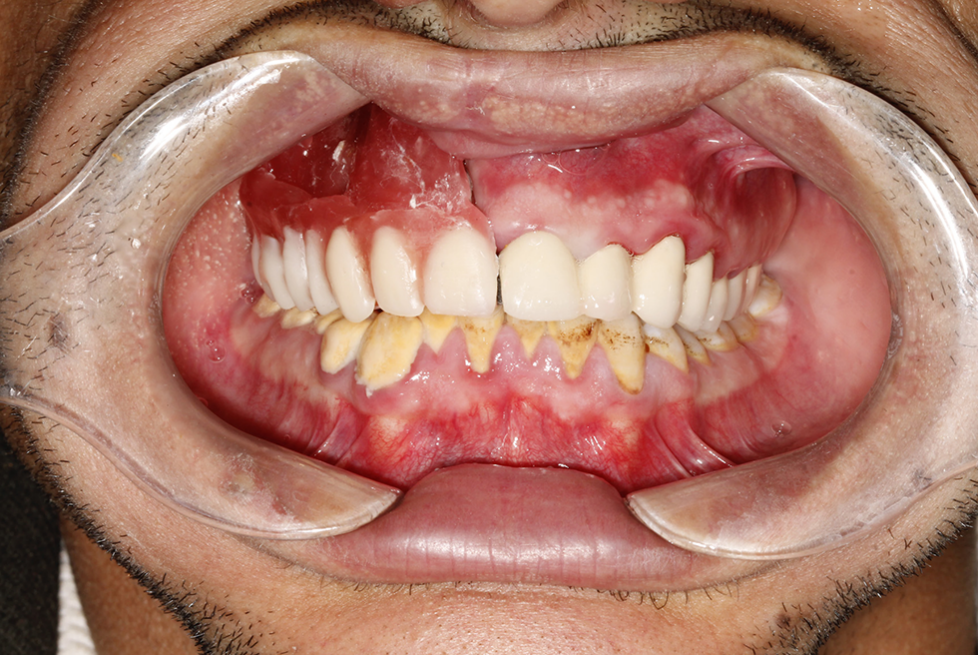
Try in of waxed obturator.

Metal group: Blocking out under the attachment with wax was also performed, and the areas around the gingival margins were relieved, then duplication of the cast was performed. The wax pattern for the obturator was made as a complete palatal plate extending to the posterior palatal borders and resting on the ledge of splinted crowns and a meshwork over the defective area approximately 3-mm short of the peripheral tissues. Casting, finishing, and polishing the framework was performed except for the inner surface of the attachment, which was sandblasted only. The clips of light retention were placed using a clip inserting tool inside the attachment space. The framework was tried in the patient’s mouth and checked for adaptation and extension ( Fig. 5 ). After the metal framework try-in, the setting up of artificial teeth and waxing-up were performed then the obturator was tried in the patient’s mouth ( Fig. 6 ). Care was taken during the final try-in to ensure restoration of oronasal separation. Then, the construction of the definitive obturator was completed.

Conventional group: The design of the definitive obturator included double Aker’s clasp on the first and second premolars and molars with alternating buccal and lingual retention, palatal plate as a major connector, and a meshwork extension at the defect side. After mouth preparation, the maxillary final impression was made using a medium body rubber base in a custom tray. The impression was then poured to obtain the master cast. After duplication, construction of the metal framework and metal framework try-in was performed. Setting up artificial teeth and waxing up was performed and tried in the patient’s mouth. Then, the construction of the definitive obturator was completed with a hard resin. Functional relining of all obturators was made with Soft silicone liner (Coe-Soft Professional Package, GC) to improve the comfort and adaptation of the obturators.

Radiographic evaluation: For ensuring standardization of measurements, digital radiographs were taken using a long-cone paralleling technique with file holder (Rinn XCP) at the time of prosthesis insertion and 6, 9, and 12 months after prosthesis insertion.

Patient satisfaction: The questionnaires were recorded 1 week, 3 months, and 6 months after prosthesis insertion for patients of all groups. All questionnaires were taken by the same research interviewer (assisted interviewer) as he was blind about the type of prosthesis and was from another department.

Two scales were followed in this study which are:

1: The Obturator Functioning Scale.

2: The European Organization for Research and Treatment of Cancer Head and Neck 35.

The questionnaire number 29 and 30 (items related to sexual life) were excluded due to social traditions. All questionnaires were in English form and translated during the interview.

Numerical data were explored for normality by checking the distribution of data and using tests of normality (Kolmogorov–Smirnov and Shapiro–Wilk tests). All data showed normal (parametric) distribution. Data were presented as mean, standard deviation (SD), and 95% confidence interval for the mean (95% CI) values. Repeated measures one-way ANOVA test was used to compare the groups and study the changes by time within each group. Bonferroni’s post-hoc test was used for pairwise comparisons. The significance level was set at *p* <0.05. Statistical analysis was performed with IBM SPSS Statistics for Windows, version 23.0 (IBM Corp.).

## Results

The data were collected for all participants along the follow-up periods with no dropout.

### Obturator Functional Scale

Both the PEEK and metal groups showed a statistically significant decrease score ( *p* <0.050) as compared with the conventional group with respect to satisfaction with the look and noticeable clasps along all follow-up periods while regarding difficulty talking to the public, both the PEEK and metal groups showed a statistically significant decrease score ( *p* <0.050) as compared with the conventional group at 1 week and 3 months. Both the PEEK and metal groups showed a statistically significant decrease score ( *p* <0.050) compared with the conventional group with regard to the difficulty of obturator insertion at the time of prosthesis insertion. The PEEK group showed a statistically significant decrease score ( *p* <0.050) than the metal group with regard to the satisfaction with the look along all follow-up periods ( Table 1 ).

**Table 1:** Comparison between the obturator functional scale scores in the three groups after 1 wk, 3 mo, and 6 mo

		Time of evaluation post insertion	PEEK		Metal		Clasp		*p* -Value between PEEK and metal	95% confidence interval PEEK and metal	*p* -Value between PEEK and clasp	95% confidence interval PEEK and clasp	*p* -Value between clasp and metal	95% confidence interval PEEK and clasp
			Mean	SD	Mean	SD	Mean	SD						
1	Difficult chewing	1 wk	1.125	0.353	1.250	0.462	1.375	0.517	0.552	0.3159/0.5659	0.277	0.2247/0.7247	0.618	0.4008/0.6508
1		3 mo	1.125	0.353	1.125	0.353	1.250	0.462	1.00	0.3786/0.3786	0.552	0.3159/0.5659	0.552	0.3159/0.5659
1		6 mo	1.00	0.000	1.00	0.000	1.250	0.462	1.000	0.0000/0.0000	0.148	0.6003/0.1003	0.148	0.6003/0.1003
2	Leakage of swallowed liquids	1 wk	1.125	0.353	1.125	0.353	1.125	0.353	1.00	0.3786/0.3786	1.00	0.3786/0.3786	1.00	0.3786/0.3786
2		3 mo	1.00	0.000	1.00	0.000	1.00	0.000	1.0000	0.0000/0.0000	1.0000	0.0000/0.0000	1.0000	0.0000/0.0000
2		6 mo	1.125	0.353	1.00	0.000	1.125	0.353	0.333	0.1427/0.3927	1.00	0.3786/0.3786	0.333	0.1427/0.3927
3	Leakage of swallowed food	1 wk	1.00	0.000	1.00	0.000	1.0	0.000	1.0000	0.0000/0.0000	1.0000	0.0000/0.0000	1.0000	0.0000/0.0000
3		3 mo	1.00	0.000	1.00	0.000	1.00	0.000	1.0000	0.0000/0.0000	1.0000	0.0000/0.0000	1.0000	0.0000/0.0000
3		6 mo	1.00	0.000	1.00	0.000	1.00	0.000	1.0000	0.0000/0.0000	1.0000	0.0000/0.0000	1.0000	0.0000/0.0000
4	Voice difference	1 wk	1.50	0.534	1.50	0.534	1.75	0.462	1.000	0.5727/0.5727	0.333	0.2854/0.7854	0.333	0.2854/0.7854
4		3 mo	1.125	0.353	1.125	0.353	1.125	0.353	1.00	0.3786/0.3786	1.00	0.3786/0.3786	1.00	0.3786/0.3786
4		6 mo	1.125	0.353	1.125	0.353	1.125	0.353	1.00	0.3786/0.3786	1.00	0.3786/0.3786	1.00	0.3786/0.3786
5	Nasal speech	1 wk	1.250	0.462	1.250	0.462	1.250	0.462	1.000	0.4954/0.4954	1.000	0.4954/0.4954	1.000	0.4954/0.4954
5		3 mo	1.250	0.462	1.250	0.462	1.250	0.462	1.000	0.4954/0.4954	1.000	0.4954/0.4954	1.000	0.4954/0.4954
5		6 mo	1.125	0.353	1.125	0.353	1.125	0.353	1.00	0.3786/0.3786	1.00	0.3786/0.3786	1.00	0.3786/0.3786
6	Difficult pronunciation	1 wk	1.375	0.517	1.375	0.517	1.625	0.571	1.000	0.5544/0.5544	0.374	0.3341/0.8341	0.374	0.3341/0.8341
6		3 mo	1.250	0.462	1.250	0.462	1.250	0.462	1.000	0.4954/0.4954	1.000	0.4954/0.4954	1.000	0.4954/0.4954
6		6 mo	1.125	0.353	1.125	0.353	1.125	0.353	1.00	0.3786/0.3786	1.00	0.3786/0.3786	1.00	0.3786/0.3786
7	Speech is difficult/be understood	1 wk	1.250	0.462	1.250	0.462	1.375	0.517	1.000	0.4954/0.4954	0.618	0.4008/0.6508	0.618	0.4008/0.6508
7		3 mo	1.125	0.353	1.125	0.353	1.125	0.353	1.00	0.3786/0.3786	1.00	0.3786/0.3786	1.00	0.3786/0.3786
7		6 mo	1.00	0.000	1.00	0.000	1.00	0.000	1.0000	0.0000/0.0000	1.0000	0.0000/0.0000	1.0000	0.0000/0.0000
8	Difficulty of talking/public	1 wk	1.25 ^A^	0.462	1.25 ^A^	0.462	2.00 ^BD^	0.5	1.000	0.4954/0.4954	0.007 ^a^	1.2662/0.2338	0.007 ^a^	1.2662/0.2338
8		3 mo	1.125	0.353	1.125	0.353	1.625 ^B^	0.774	1.00	0.3786/0.3786	0.118	1.1451/0.1451	0.118	1.1451/0.1451
8		6 mo	1.125	0.353	1.125	0.353	1.375 ^E^	0.517	1.00	0.3786/0.3786	0.277	0.7247/0.2247	0.277	0.7247/0.2247
9	Dry mouth	1 wk	1.5	0.534	1.5	0.534	1.5	0.534	1.000	0.5727/0.5727	1.000	0.5727/0.5727	1.000	0.572/0.5727
9		3 mo	1.5	0.534	1.5	0.534	1.5	0.534	1.000	0.5727/0.5727	1.000	0.5727/0.5727	1.000	0.5727/0.5727
9		6 mo	1.5	0.534	1.5	0.534	1.5	0.534	1.000	0.5727/0.5727	1.000	0.5727/0.5727	1.000	0.5727/0.5727
10	Satisfaction with look	1 wk	1.00 ^A^	0.000	1.50 ^B^	0.534	2.25 ^C^	0.707	0.019 ^a^	0.0951/0.9049	0.0002 ^a^	0.7139/1.7861	0.031 ^a^	0.0781/1.4219
10		3 mo	1.00 ^A^	0.000	1.50 ^B^	0.534	2.25 ^C^	0.707	0.019 ^a^	0.0951/0.9049	0.0002 ^a^	0.7139/1.7861	0.031 ^a^	0.0781/1.4219
10		6 mo	1.00 ^A^	0.000	1.50 ^B^	0.534	2.25 ^C^	0.707	0.019 ^a^	0.0951/0.9049	0.0002 ^a^	0.7139/1.7861	0.031 ^a^	0.0781/1.4219
11	Noticeable clasps	1 wk	1.00 ^A^	0.000	1.00 ^A^	0.000	2.25 ^B^	0.707	0.0002 ^a^	0.7139/1.7861	1.0000	0.0000/0.0000	0.0002 ^a^	0.7139/1.7861
11		3 mo	1.00 ^A^	0.000	1.00 ^A^	0.000	2.25 ^B^	0.707	0.0002 ^a^	0.7139/1.7861	1.0000	0.0000/0.0000	0.0002 ^a^	0.7139/1.7861
11		6 mo	1.00 ^A^	0.000	1.00 ^A^	0.000	2.25 ^B^	0.707	0.0002 ^a^	0.7139/1.7861	1.0000	0.0000/0.0000	0.0002 ^a^	0.7139/1.7861
12	Numb lips	1 wk	1.00	0.000	1.00	0.000	1.00	0.000	1.000	0.0000/0.0000	1.0000	0.0000/0.0000	1.0000	0.0000/0.0000
12		3 mo	1.00	0.000	1.00	0.000	1.00	0.000	1.000	0.0000/0.0000	1.0000	0.0000/0.0000	1.0000	0.0000/0.0000
12		6 mo	1.00	0.000	1.00	0.000	1.00	0.000	1.000	0.0000/0.0000	1.0000	0.000/0.0000	1.0000	0.0000/0.0000
13	Lips look funny	1 wk	1.00	0.000	1.00	0.000	1.00	0.000	1.000	0.0000/0.0000	1.0000	0.0000/0.0000	1.0000	0.0000/0.0000
13		3 mo	1.00	0.000	1.00	0.000	1.00	0.000	1.000	0.0000/0.0000	1.0000	0.0000/0.0000	1.0000	0.0000/0.0000
13		6 mo	1.00	0.000	1.00	0.000	1.00	0.000	1.000	0.0000/0.0000	1.0000	0.0000/0.0000	1.0000	0.000/0.0000
14	Avoidance of family social events	1 wk	1.25	0.462	1.375	0.517	1.5	0.534	0.618	0.4008/0.6508	0.333	0.285/0.7854	0.641	0.4386/0.6886
14		3 mo	1.125	0.353	1.125	0.353	1.375	0.517	1.00	0.3786/0.3786	0.277	0.7247/0.2247	0.277	0.7247/0.2247
14		6 mo	1.00	0.000	1.125	0.353	1.125	0.353	0.333	0.3927/0.1427	0.333	0.3927/0.1427	1.00	0.3786/0.3786
15	Difficult insertion of obturator	1 wk	1.125 ^A^	0.353	1.125 ^A^	0.353	1.875 ^BD^	0.834	1.00	0.3786/0.3786	0.034 ^a^	0.0633/1.4367	0.034	0.0633/1.4367
15		3 mo	1.125	0.353	1.125	0.353	1.50	0.534	1.00	0.3786/0.3786	0.119	0.1104/0.8604	0.119	0.1104/0.8604
15		6 mo	1.00	0.000	1.00	0.000	1.125 ^E^	0.408	1.000	0.0000/0.0000	0.333	0.3927/0.1427	0.333	0.3927/0.1427
16	Overall score	1 wk	17.83	4.382	18.33	4.806	22.17	5.933	0.831	4.4318/5.4318	0.118	1.2530/9.9330	0.176	1.9498/9.6298
16		3 mo	16.83	2.858	17.33	2.658	20.33	3.017	0.722	2.4596/3.4596	0.031 ^a^	0.3487/6.6513	0.053	0.0490/6.0490
16		6 mo	16.33	2.805	17.03	3.521	19.33	3.830	0.666	2.7136/4.1136	0.095	0.5999/6.5999	0.231	1.6451/6.2451
Abbreviation: PEEK, polyetheretherketone. ^a^ Significant at *p* ≤ 0.05.														

### The European Organization for Research and Treatment of Cancer Head and Neck 35

Both the PEEK and metal groups showed a statistically significant decrease score ( *p* <0.050) as compared with the conventional group with respect to the satisfaction of appearance along all follow-up periods. While with regard to talking to public, both the PEEK and metal groups showed a statistically significant decrease score ( *p* <0.050) as compared with the conventional group at insertion and at 3 months. Both the PEEK and metal groups showed a statistically significant decrease score ( *p* <0.050) than the conventional group regarding eating in front of people at insertion. The PEEK and metal groups showed a statistically significant decrease score ( *p* <0.050) than the conventional group with respect to gaining weight at 3 months. The PEEK group showed a statistically significant decrease score ( *p* <0.050) than the metal group with regard to satisfaction with the look along all follow-up periods ( Table 2 ).

**Table 2:** Comparison between the mean EORTC QLQ—H&N35 scores in the three groups after 1 wk, 3 mo and 6 mo

	Have you had	Time of evaluation post insertion	PEEK		Metal		Clasp		*p* -Value between and 95% confidence interval					
			Mean	SD	Mean	SD	Mean	SD	PEEK and metal 95% CI	p-Value betweenPEEK and metal	PEEK and Clasp 95% CI	p-Value betweenPEEK and clasp	*Metal and clasp* 95% CI	*p-Value betweenMetal and clasp*
1	Pain in your mouth?	1 wk	1.375	0.517	1.5	0.534	1.5	0.534	0.4386 to 0.6886	0.641	0.4386 to 0.6886	0.641	0.5727 to 0.5727	1.000
1		3 mo	1. 25	0.462	1. 25	0.462	1. 25	0.462	0.3786 to 0.3786	1.00	0.3786 to 0.3786	1.00	0.3786 to 0.3786	1.00
1		6 mo	1.125	0.353	1.125	0.353	1.125	0.353	0.4954 to 0.4954	1.000	0.4954 to 0.4954	1.000	0.4954 to 0.4954	1.000
2	Pain in your jaw?	1 wk	1.375	0.517	1.375	0.517	1.375	0.517	0.5544 to 0.5544	1.000	0.5544 to 0.5544	1.000	0.5544 to 0.5544	1.000
2		3 mo	1.375	0.517	1.375	0.517	1.375	0.517	0.5544 to 0.5544	1.000	0.5544 to 0.5544	1.000	0.5544 to 0.5544	1.000
2		6 mo	1.375	0.517	1.375	0.517	1.375	0.517	-0.5544 to 0.5544	1.000	0.5544 to 0.5544	1.000	0.5544 to 0.5544	1.000
3	Soreness in your mouth?	1 wk	1.5	0.534	1.5	0.534	1.5	0.534	0.5727 to 0.5727	1.000	0.5727 to 0.5727	1.000	0.5727 to 0.5727	1.000
3		3 mo	1.375	0.517	1.375	0.517	1.375	0.517	0.5544 to 0.5544	1.000	0.5544 to 0.5544	1.000	0.5544 to 0.5544	1.000
3		6 mo	1.375	0.517	1.375	0.517	1.375	0.517	0.5544 to 0.5544	1.000	0.5544 to 0.5544	1.000	0.5544 to 0.5544	1.000
4	A painful throat?	1 wk	1.0	0.0	1.0	0.0	1.0	0.0	0.0000 to 0.0000	1.000	0.0000 to 0.0000	1.000	0.0000 to 0.0000	1.000
4		3 mo	1.0	0.0	1.0	0.0	1.0	0.0	0.0000 to 0.0000	1.000	0.0000 to 0.0000	1.000	0.0000 to 0.0000	1.000
4		6 mo	1.0	0.0	1.0	0.0	1.0	0.0	0.0000 to 0.0000	1.000	0.0000 to 0.0000	1.000	0.0000 to 0.0000	1.000
5	Problems swallowing liquids?	1 wk	1.125	0.353	1.125	0.353	1.125	0.353	0.3786 to 0.3786	1.00	0.3786 to 0.3786	1.00	0.3786 to 0.3786	1.00
5		3 mo	1.0	0.0	1.0	0.0	1.125	0.353	0.0000 to 0.0000	1.000	0.1427 to 0.3927	0.333	0.1427 to 0.3927	0.333
5		6 mo	1.125	0.353	1.0	0.0	1.125	0.353	0.1427 to 0.3927	0.333	0.3786 to 0.3786	1.00	0.1427 to 0.3927	0.333
6	Problems swallowing pureed food?	1 wk	1.125	0.353	1.125	0.353	1.125	0.353	0.3786 to 0.3786	1.00	0.3786 to 0.3786	1.00	0.3786 to 0.3786	1.00
6		3 mo	1.0	0.0	1.0	0.0	1.125	0.353	0.0000 to 0.0000	1.000	0.1427 to 0.3927	0.333	0.1427 to 0.3927	0.333
6		6 mo	1. 0	0.00	1.0	0.0	1.125	0.353	0.0000 to 0.0000	1.000	-0.1427 to 0.3927	0.333	-0.1427 to 0.3927	0.333
7	Problems swallowing solid food?	1 wk	1.0	0.0	1.0	0.0	1.0	0.0	0.0000 to 0.0000	1.000	0.0000 to 0.0000	1.000	0.0000 to 0.0000	1.000
7		3 mo	1.0	0.0	1.0	0.0	1.0	0.0	0.0000 to 0.0000	1.000	0.0000 to 0.0000	1.000	0.0000 to 0.0000	1.000
7		6 mo	1.0	0.0	1.0	0.0	1.0	0.0	0.0000 to 0.0000	1.000	0.0000 to 0.0000	1.000	0.0000 to 0.0000	1.000
8	Have you choked when swallowing?	1 wk	1.25	0.462	1.25	0.462	1.25	0.462	0.4954 to 0.4954	1.000	0.4954 to 0.4954	1.000	0.4954 to 0.4954	1.000
8		3 mo	1.0	0.0	1.0	0.0	1.0	0.0	0.0000 to 0.0000	1.000	0.0000 to 0.0000	1.000	0.0000 to 0.0000	1.000
8		6 mo	1.0	0.0	1.0	0.0	1.0	0.0	0.0000 to 0.0000	1.000	0.0000 to 0.0000	1.000	0.0000 to 0.0000	1.000
9	problems with your teeth?	1 wk	1.125	0.353	1.125	0.353	1.375	0.517	0.3786 to 0.3786	1.00	0.2247 to 0.7247	0.277	0.2247 to 0.7247	0.277
9		3 mo	1.00	0.0	1.00	0.0	1.375	0.517	0.0000 to 0.0000	1.000	0.0170 to 0.7670	0.059	0.0170 to 0.7670	0.059
9		6 mo	1.00	0.0	1.00	0.0	1.375	0.517	0.0000 to 0.0000	1.000	0.0170 to 0.7670	0.059	0.0170 to 0.7670	0.059
10	Problems opening your mouth wide?	1 wk	1.625	0.774	1.875	0.834	1.75	0.886	0.6128 to 1.1128	0.544	0.7671 to 1.0171	0.768	1.0477 to 0.7977	0.775
10		3 mo	1.5	0.534	1.5	0.534	1.5	0.534	0.5727 to 0.5727	1.000	0.5727 to 0.5727	1.000	0.5727 to 0.5727	1.000
10		6 mo	1.5	0.534	1.375	0.517	1.375	0.517	0.4386 to 0.6886	0.641	0.4386 to 0.6886	0.641	0.5544 to 0.5544	1.000
11	A dry mouth?	1 wk	1.5	0.534	1.5	0.534	1.5	0.534	0.5727 to 0.5727	1.000	0.5727 to 0.5727	1.000	0.5727 to 0.5727	1.000
11		3 mo	1.5	0.534	1.5	0.534	1.5	0.534	0.5727 to 0.5727	1.000	0.5727 to 0.5727	1.000	0.5727 to 0.5727	1.000
11		6 mo	1.5	0.534	1.5	0.534	1.5	0.534	0.5727 to 0.5727	1.000	0.5727 to 0.5727	1.000	0.5727 to 0.5727	1.000
12	sticky saliva?	1 wk	1.5	0.534	1.5	0.534	1.5	0.534	0.5727 to 0.5727	1.000	0.5727 to 0.5727	1.000	0.5727 to 0.5727	1.000
12		3 mo	1.5	0.534	1.5	0.534	1.5	0.534	0.5727 to 0.5727	1.000	0.5727 to 0.5727	1.000	0.5727 to 0.5727	1.000
12		6 mo	1.5	0.534	1.5	0.534	1.5	0.534	0.5727 to 0.5727	1.000	0.5727 to 0.5727	1.000	0.5727 to 0.5727	1.000
13	problems with your sense of smell?	1 wk	1.0	0.0	1.0	0.0	1.0	0.0	0.0000 to 0.0000	1.000	0.0000 to 0.0000	1.000	0.0000 to 0.0000	1.000
13		3 mo	1.0	0.0	1.0	0.0	1.0	0.0	0.0000 to 0.0000	1.000	0.0000 to 0.0000	1.000	0.0000 to 0.0000	1.000
13		6 mo	1.0	0.0	1.0	0.0	1.0	0.0	0.0000 to 0.0000	1.000	0.0000 to 0.0000	1.000	0.0000 to 0.0000	1.000
14	Problems with your sense of taste?	1 wk	1.0	0.0	1.0	0.0	1.0	0.0	0.0000 to 0.0000	1.000	0.0000 to 0.0000	1.000	0.0000 to 0.0000	1.000
14		3 mo	1.0	0.0	1.0	0.0	1.0	0.0	0.0000 to 0.0000	1.000	0.0000 to 0.0000	1.000	0.0000 to 0.0000	1.000
14		6 mo	1.0	0.0	1.0	0.0	1.0	0.0	0.0000 to 0.0000	1.000	0.0000 to 0.0000	1.000	0.0000 to 0.0000	1.000
15	Have you coughed?	1 wk	1.25	0.462	1.125	0.353	1.25	0.462	0.3159 to 0.5659	0.552	0.4954 to 0.4954	1.000	0.3159 to 0.5659	0.552
15		3 mo	1.0	0.0	1.0	0.0	1.0	0.0	0.0000 to 0.0000	1.000	0.0000 to 0.0000	1.000	0.0000 to 0.0000	1.000
15		6 mo	1.0	0.0	1.0	0.0	1.0	0.0	0.0000 to 0.0000	1.000	0.0000 to 0.0000	1.000	0.0000 to 0.0000	1.000
16	Have you been hoarse?	1 wk	1.375	0.517	1.375	0.517	1.375	0.517	0.5544 to 0.5544	1.000	0.5544 to 0.5544	1.000	0.5544 to 0.5544	1.000
16		3 mo	1.0	0.0	1.0	0.0	1.0	0.0	0.0000 to 0.0000	1.000	0.0000 to 0.0000	1.000	0.0000 to 0.0000	1.000
16		6 mo	1.0	0.0	1.0	0.0	1.0	0.0	0.0000 to 0.0000	1.000	0.0000 to 0.0000	1.000	0.0000 to 0.0000	1.000
17	Have you felt ill?	1 wk	1.375	0.517	1.375	0.517	1.375	0.517	0.5544 to 0.5544	1.000	0.5544 to 0.5544	1.000	0.5544 to 0.5544	1.000
17		3 mo	1.375	0.517	1.375	0.517	1.375	0.517	0.5544 to 0.5544	1.000	0.5544 to 0.5544	1.000	0.5544 to 0.5544	1.000
17		6 mo	1.375	0.517	1.375	0.517	1.375	0.517	0.5544 to 0.5544	1.000	0.5544 to 0.5544	1.000	0.5544 to 0.5544	1.000
18	Has your appearance bothered you?	1 wk	1.0 ^A^	0.0	1.5 ^B^	0.534	2.25 ^C^	0.707	0.0951 to 0.9049	0.019 ^a^	0.7139 to 1.7861	0.0002 ^a^	0.0781 to 1.4219	0.031 ^a^
18		3 mo	1.0 ^A^	0.0	1.5 ^B^	0.534	2.25 ^C^	0.707	0.0951 to 0.9049	0.019 ^a^	0.7139 to 1.7861	0.0002 ^a^	0.0781 to 1.4219	0.031 ^a^
18		6 mo	1.0 ^A^	0.0	1.5 ^B^	0.534	2.25 ^C^	0.707	0.0951 to 0.9049	0.019 ^a^	0.7139 to 1.7861	0.0002 ^a^	0.0781 to 1.4219	0.031 ^a^
19	Trouble eating?	1 wk	1.375	0.517	1.375	0.517	1.375	0.517	0.5544 to 0.5544	1.000	0.5544 to 0.5544	1.000	0.5544 to 0.5544	1.000
19		3 mo	1.125	0.353	1.125	0.353	1.375	0.517	0.3786 to 0.3786	1.00	0.2247 to 0.7247	0.277	0.2247 to 0.7247	0.277
19		6 mo	1.0	0.0	1.0	0.0	1.125	0.353	0.0000 to 0.0000	1.000	0.1427 to 0.3927	0.333	0.1427 to 0.3927	0.333
20	Trouble eating in front of your family?	1 wk	1.375	0.517	1.375	0.517	1.375	0.517	0.5544 to 0.5544	1.000	0.5544 to 0.5544	1.000	0.5544 to 0.5544	1.000
20		3 mo	1.375	0.517	1.375	0.517	1.375	0.517	0.5544 to 0.5544	1.000	0.5544 to 0.5544	1.000	0.5544 to 0.5544	1.000
20		6 mo	1.0	0.0	1.0	0.0	1.125	0.353	0.0000 to 0.0000	1.000	0.1427 to 0.3927	0.333	0.1427 to 0.3927	0.333
21	Trouble eating in front of other people?	1 wk	1.375 ^A^	0.517	1.375 ^A^	0.517	2.00 ^B^	0.500	0.5544 to 0.5544	1.000	0.0796 to 1.1704	0.027 ^a^	0.0796 to 1.1704	0.027 ^a^
21		3 mo	1.375	0.517	1.375	0.517	1.625	0.571	0.5544 to 0.5544	1.000	0.3341 to 0.8341	0.374	0.3341 to 0.8341	0.374
21		6 mo	1.125	0.353	1.125	0.353	1.5	0.534	0.3786 to 0.3786	1.00	0.1104 to 0.8604	0.119	0.1104 to 0.8604	0.119
22	Trouble enjoying your meals?	1 wk	1.5	0.534	1.5	0.534	1.5	0.534	0.5727 to 0.5727	1.000	0.5727 to 0.5727	1.000	0.5727 to 0.5727	1.000
22		3 mo	1.5	0.534	1.5	0.534	1.5	0.534	0.5727 to 0.5727	1.000	0.5727 to 0.5727	1.000	0.5727 to 0.5727	1.000
22		6 mo	1.5	0.534	1.5	0.534	1.5	0.534	0.5727 to 0.5727	1.000	0.5727 to 0.5727	1.000	0.5727 to 0.5727	1.000
23	Trouble talking to other people?	1 wk	1.375 ^A^	0.517	1.375 ^A^	0.517	2.00 ^Bd^	0.5	0.5544 to 0.5544	1.000	0.0796 to 1.1704	0.027 ^a^	0.0796 to 1.1704	0.027 ^a^
23		3 mo	1.125 ^A^	0.353	1.125 ^A^	0.353	1.75 ^B^	0.886	0.3786 to 0.3786	1.00	0.0982 to 1.3482	0.085	0.0982 to 1.3482	0.085
23		6 mo	1.125	0.353	1.125	0.353	1.375 ^E^	0.517	0.3786 to 0.3786	1.00	0.2247 to 0.7247	0.277	0.2247 to 0.7247	0.277
24	Trouble talking on the teleph	1 wk	1.0	0.0	1.0	0.0	1.0	0.0	0.0000 to 0.0000	1.000	0.0000 to 0.0000	1.000	0.0000 to 0.0000	1.000
24		3 mo	1.0	0.0	1.0	0.0	1.0	0.0	0.0000 to 0.0000	1.000	0.0000 to 0.0000	1.000	0.0000 to 0.0000	1.000
24		6 mo	1.0	0.0	1.0	0.0	1.0	0.0	0.0000 to 0.0000	1.000	0.0000 to 0.0000	1.000	0.0000 to 0.0000	1.000
25	Trouble having social contact with your family?	1 wk	1.375	0.517	1.375	0.517	1.5	0.534	0.5544 to 0.5544	1.000	0.4386 to 0.6886	0.641	0.4386 to 0.6886	0.641
25		3 mo	1.125	0.353	1. 25	0.462	1.375	0.517	0.3159 to 0.5659	0.552	0.2247 to 0.7247	0.277	0.4008 to 0.6508	0.618
25		6 mo	1.0	0.0	1.125	0.353	1.125	0.353	0.1427 to 0.3927	0.333	0.1427 to 0.3927	0.333	0.3786 to 0.3786	1.00
26	Trouble having social contact with friends?	1 wk	1.375	0.517	1.375	0.517	1.5	0.534	0.5544 to 0.5544	1.000	0.4386 to 0.6886	0.641	0.4386 to 0.6886	0.641
26		3 mo	1.375	0.517	1.375	0.517	1.375	0.517	0.5544 to 0.5544	1.000	0.5544 to 0.5544	1.000	0.5544 to 0.5544	1.000
26		6 mo	1.125	0.353	1.125	0.353	1.375	0.517	0.3786 to 0.3786	1.00	0.2247 to 0.7247	0.277	0.2247 to 0.7247	0.277
27	Trouble going out in public?	1 wk	1.125	0.353	1.125	0.353	1.375	0.517	0.3786 to 0.3786	1.00	0.2247 to 0.7247	0.277	0.2247 to 0.7247	0.277
27		3 mo	1.0	0.0	1.125	0.353	1.125	0.353	0.1427 to 0.3927	0.333	0.1427 to 0.3927	0.333	0.3786 to 0.3786	1.00
27		6 mo	1.0	0.0	1.125	0.353	1.125	0.353	0.1427 to 0.3927	0.333	0.1427 to 0.3927	0.333	0.3786 to 0.3786	1.00
28	Trouble having physical contact with your family or friends?	1 wk	1.25 ^A^	0.462	1.25 ^A^	0.462	1.875 ^B^	0.640	0.4954 to 0.4954	1.000	0.0265 to 1.2235	0.041 ^a^	0.0265 to 1.2235	0.041 ^a^
28		3 mo	1.125 ^A^	0.353	1.125 ^A^	0.353	1.75 ^B^	0.462	0.3786 to 0.3786	1.00	0.1841 to 1.0659	0.008 ^a^	0.1841 to 1.0659	0.008 ^a^
28		6 mo	1	0.0	1.125	0.353	1.5	0.534	0.1427 to 0.3927	0.333	0.0951 to 0.9049	0.019 ^a^	0.1104 to 0.8604	0.119
31	Have you used painkillers?	1 wk	1.0	0.0	1.0	0.0	1.0	0.0	0.0000 to 0.0000	1.000	0.0000 to 0.0000	1.000	0.0000 to 0.0000	1.000
31		3 mo	1.0	0.0	1.0	0.0	1.0	0.0	0.0000 to 0.0000	1.000	0.0000 to 0.0000	1.000	0.0000 to 0.0000	1.000
31		6 mo	1.0	0.0	1.0	0.0	1.0	0.0	0.0000 to 0.0000	1.000	0.0000 to 0.0000	1.000	0.0000 to 0.0000	1.000
32	Have you taken any nutritional supplements (excluding vitamins)?	1 wk	1.0	0.0	1.0	0.0	1.0	0.0	0.0000 to 0.0000	1.000	0.0000 to 0.0000	1.000	0.0000 to 0.0000	1.000
32		3 mo	1.0	0.0	1.0	0.0	1.0	0.0	0.0000 to 0.0000	1.000	0.0000 to 0.0000	1.000	0.0000 to 0.0000	1.000
32		6 mo	1.0	0.0	1.0	0.0	1.0	0.0	0.0000 to 0.0000	1.000	0.0000 to 0.0000	1.000	0.0000 to 0.0000	1.000
33	Have you used a feeding tube?	1 wk	1.0	0.0	1.0	0.0	1.0	0.0	0.0000 to 0.0000	1.000	0.0000 to 0.0000	1.000	0.0000 to 0.0000	1.000
33		3 mo	1.0	0.0	1.0	0.0	1.0	0.0	0.0000 to 0.0000	1.000	0.0000 to 0.0000	1.000	0.0000 to 0.0000	1.000
33		6 mo	1.0	0.0	1.0	0.0	1.0	0.0	0.0000 to 0.0000	1.000	0.0000 to 0.0000	1.000	0.0000 to 0.0000	1.000
34	Have you lost weight?	1 wk	1.0	0.0	1.0	0.0	1.0	0.0	0.0000 to 0.0000	1.000	0.0000 to 0.0000	1.000	0.0000 to 0.0000	1.000
34		3 mo	1.0	0.0	1.0	0.0	1.0	0.0	0.0000 to 0.0000	1.000	0.0000 to 0.0000	1.000	0.0000 to 0.0000	1.000
34		6 mo	1.0	0.0	1.0	0.0	1.0	0.0	0.0000 to 0.0000	1.000	0.0000 to 0.0000	1.000	0.0000 to 0.0000	1.000
35	Have you gained weight?	1 wk	1.0	0.0	1.0	0.0	1.0	0.0	0.0000 to 0.0000	1.000	0.0000 to 0.0000	1.000	0.0000 to 0.0000	1.000
35		3 mo	1.625	0.571	1.875	0.834	1.625	0.571	0.5164 to 1.0164	0.495	0.6123 to 0.6123	1.000	0.5164 to 1.0164	0.495
35		6 mo	1.0	0.0	1.0	0.0	1.0	0.0	0.0000 to 0.0000	1.000	0.0000 to 0.0000	1.000	0.0000 to 0.0000	1.000
Abbreviation: PEEK, polyetheretherketone. ^a^ Significant at *p* ≤0.05. ^A, B, C^ in the same row indicate statistically significant difference between groups. ^D,E^ ,F in the same column indicate statistically significant change by time.														

### Radiographic Evaluation

Both PEEK and metal groups showed a statistically significant lower mean bone loss than the conventional group during all follow-up periods. There was no statistically significant increase in the mean amount of bone loss within all groups from 6 to 9 months as well as from 6 to 12 months. There is no statistically significant difference between the PEEK group and the metal group during all follow-up periods ( Table 3 ).

**Table 3:** Comparison between bone height measurements in the three groups at 6, 9 and 12 mo

	PEEK		Metal		Clasp		*p* -Value between all groups			95% confidence interval		
	Mean	SD	Mean	SD	Mean	SD	PEEK and clasp	Clasp and metal	PEEK and meta	PEEK and metal	PEEK and clasp	Clasp and metal
Radio-6m	0.2250	0.01761	0.2333	0.02251	0.3650	0.05753	0.0001 ^a^	0.0001 ^a^	0.4252	0.0300/0.0134	0.0944/0.1856	0.0849/0.1785
Radio-9m	0.1000	0.01414	0.1050	0.01049	0.1083	0.01472	0.2694	0.6136	0.4353	0.0084/0.0184	0.0072/0.0238	0.0104/0.0170
Radio-12m	0.0550	0.03146	0.0600	0.05177	0.0633	0.03882	0.6457	0.8874	0.8188	0.0409/0.0509	0.0296/0.0462	0.0458/0.0524
Abbreviation: PEEK, polyetheretherketone. ^a^ Significant at *p* ≤0.05.												

## Discussion

The various obturators were mainly constructed for rehabilitating maxillary defects. Well-designed obturators were made to provide durable and excellent retention, stability, and support and improve patient satisfaction and quality of life. One of the essential keys to obturator success is the retention of prosthesis. 29 42 43 44 45

The use of PEEK as an alternative to the metal is excellent, which supports the study hypothesis. All patients of the three groups were satisfied with their definitive obturators but with different degrees. The satisfaction started from prosthesis insertion and increased gradually till the end of the study period. The patients of both the PEEK and metal groups were relatively more satisfied than the conventional group. 42 44

In the past, the absence of validated questionnaires was one of the major problems in evaluating the QOL and satisfaction of maxillofacial prosthesis wearers, but now there are several validated questionnaires. 43 46 47

Satisfaction with look and appearance in the PEEK and metal groups was markedly improved compared with the conventional group. This may be attributed to the absence of clasp, improving the gingival architecture during the teeth preparations, selecting a lighter shade of porcelain crowns, as well as a lighter shade of acrylic teeth in the metal group and “Crea.lign” veneering (Bredent GmbH & Co. KG, Senden, Germany) for teeth in the PEEK group which showed superior esthetics. In the conventional group, a noticeable clasp was a negative point that annoyed the patients. The shade and form of the teeth are not bright and white as it is governed by the shade and the form of the remaining natural teeth on the intact side. The unpleasant appearance of the anterior teeth in the obturator was apparent during smiling. 30 31 42 44

The speech was generally relatively improved in all groups, better in both PEEK and metal groups than the conventional group, as the function of the obturator was enhanced when adding an attachment, especially speech. 29 42 This may be attributed to accurate fit, the adaptation of the obturator, improved retention, and stability gained by the attachments compared with clasps and feeling of comfort during the speech. While talking on the telephone without visual cues and talking in public, the former showed no difference between all groups. In contrast, the difficulty of talking in public was diminished in both PEEK and metal groups due to improved esthetics, personal confidence regarding received treatment, and no fear of social contact, and avoidance of social rejection. The peek and metal groups were more satisfied with their obturator insertion, which may be attributed to the simple insertion path compared with the conventional group.

The problems related to swallowing, leakage of liquids, leakage of pureed food, leakage of solid food, choking when swallowing, and chewing difficulty showed a slight improvement in all groups. Those problems are mainly affected by the adaptation of the obturator, the remaining structures, the remaining natural teeth, and the degree of separation between the oral and nasal cavities as incompetent separation results in the ingress of fluids and food to the nasal cavity. As the patients chew on the intact side (unresected side), not using the defect side, the improvement of obturator adaptation may be gained by functional relining material that changed periodically every 6 months. The less possible movements in both PEEK and metal groups may be a factor for efficient separation, helping the patient enjoy eating and swallowing, which is apparent in PEEK and metal groups. 29 42

Eating in front of the public, avoiding physical contact with friends, and avoiding family social events were significantly improved in both PEEK and metal groups compared with the conventional group. As which attributed to no fear of social contact, no fear of social rejection, retention, and stability of obturator helping the patient to eat and swallow hence confidence in front of anyone. Eating in front of family and enjoying meals do not differ in all groups but relatively improved with time as there is no embarrassment between family members where the weight gain improved for all groups after 6 months, which reflects the satisfaction and improved ability for eating.

As the retention of conventional obturator depends mainly on clasps engaging undercuts around the healthy abutment, the clasps have a horizontal force exerted to abutment teeth due to multiple cycles of insertion/removal, which may lead to periodontal affection and successive alveolar bone resorption, especially to the abutment teeth neighbor to the defect. Splinting of abutment teeth provides better stress distribution, especially to the abutment teeth neighbor to the defect, which reflects the alveolar bone resorption around abutment teeth. 20 42

## Limitations

Due to the height of the defect in one case, only the dimensions of the PEEK obturator were higher than the height of the blank (23 mm). Thus, the prosthesis could not be milled in proper dimensions, so modification of the palatal contour of the obturator (makes it shallower) was made to be compatible according to the blank height.

## Conclusions

In conclusion, it can be said that PEEK attachment-retained maxillary definitive obturators could be considered as a promising treatment modality for patients with acquired maxillary defects regarding esthetics and appearance satisfaction.
